# Melatonin Participates in Selenium-Enhanced Cold Tolerance of Cucumber Seedlings

**DOI:** 10.3389/fpls.2021.786043

**Published:** 2021-12-22

**Authors:** Ning Yang, Kaining Sun, Xiao Wang, Kean Wang, Xianghua Kong, Jianwei Gao, Dan Wen

**Affiliations:** ^1^Shandong Key Laboratory of Greenhouse Vegetable Biology, Huang-Huai-Hai Region Scientific Observation and Experimental Station of Vegetables, Ministry of Agriculture and Rural Affairs, Shandong Branch of National Improvement Center for Vegetables, Institute of Vegetables Research, Shandong Academy of Agricultural Sciences, Jinan, China; ^2^Key Laboratory of Plant Development and Environmental Adaption Biology, Ministry of Education, School of Life Science, Shandong University, Qingdao, China

**Keywords:** selenium, melatonin, cucumber, cold stress, *COMT*

## Abstract

Melatonin is an important and widespread plant hormone. However, the underlying physiological and molecular mechanisms of melatonin as a secondary messenger in improving cold tolerance by selenium are limited. This study investigated the effects of selenite on the cold stress of cucumber seedlings. The results showed that exogenous application of selenite improved the cold tolerance of cucumber seedlings, which was dependent on the concentration effect. In the present experiment, 1 μM of selenite showed the best effect on alleviating cold stress. Interestingly, we found that in the process of alleviating cold stress, selenite increased the content of endogenous melatonin by regulating the expression of melatonin biosynthesis genes (*TDC, T5H, SNAT*, and *COMT*). To determine the interrelation between selenite and melatonin in alleviating cold stress, melatonin synthesis inhibitor *p*-chlorophenylalanine and melatonin were used for in-depth study. This study provides a theoretical basis for cucumber cultivation and breeding.

## Introduction

Selenium was discovered by Berzelius in 1817, which plays vital roles in humans with functions in preventing cancer, boosting immunity, detoxifying cells, and even in combatting COVID-19 infections (Santi and Bagnoli, 2017; Moghaddam et al., 2020; Zhang et al., 2020). Selenium is generally beneficial for plants in appropriate concentrations and has been found to influence photosynthesis (Jiang et al., 2017), root architecture (Zhao et al., 2019), senescence (Hajiboland et al., 2019), vegetable quality (Mckenzie et al., 2019), defense, and stress response (Alves et al., 2020; Wen, 2021). With the interest in the roles of selenium in plants, reports of selenium have dramatically increased in recent years and it is anticipated that mechanism studies related to plant selenium will flourish in the near future (Wrobel et al., 2020; Tran et al., 2021; Wen, 2021).

Melatonin is an important and widespread plant hormone. In 1995, reports have demonstrated the presence of natural melatonin in the plant kingdom (Dubbels et al., 1995; Hattori et al., 1995). Scientists have conducted more comprehensive and in-depth studies on the synthesis, content, distribution, and function of melatonin in plants. Melatonin as plant master regulator plays vital roles in plant growth (Erdal, 2019), crop yield (Zahedi et al., 2020), senescence (Tan et al., 2020), storage and fresh-keeping (Onik et al., 2021), root development (Wen et al., 2016), stress response, and so on (Arnao and Hernández-Ruiz, 2019; Khan et al., 2020). Although several researchers have studied the effects of exogenous melatonin on enhancing plant cold tolerance (Li et al., 2018; Liu et al., 2020), not much is known about the mechanism of melatonin associated with selenium as well as the related signal transduction events under the condition of cold stress.

In the present study, melatonin-participated selenium enhanced plant cold tolerance of cucumber seedlings. Pharmacologic method was used in which *p*-chlorophenyl alanine (CPA) reduced melatonin biosynthesis (Murch et al., 2001; Ramakrishna et al., 2009; Park, 2011; Feng et al., 2021). Fluorescence quantitative technique was used to identify the expression of key genes in this process. Levels of melatonin biosynthesis genes *TDC* (tryptophan decarboxylase), *T5H* (tryptamine 5-hydroxylase), *SNAT* (serotonin *N*-acetyltransferase), and *COMT* (caffeic acid *O*-methyltransferase) were detected (Tan et al., 2015). *T5H*, which is the key gene that regulates the pathway from tryptamine to biosynthesis of melatonin rather than auxin (Back, 2021), significantly changed in exogenous application of selenium with cold stress of cucumber seedlings. *COMT*, as the key gene, which regulates the last step of melatonin synthesis (Byeon et al., 2015; Sun et al., 2020), also changed significantly. The present study deepens the understanding of selenium and melatonin in plant cold response as well as signaling transduction in plants.

## Materials and Methods

### Plant Materials, Growth Conditions, and Treatments

Cucumber (Jinyan No. 4) seeds were sterilized in 2.5% NaClO and washed three times for 5 min in sterile distilled water, then soaked in distilled water for 6 h at 28°C. After the seeds were germinated on filter paper in Petri dishes, they were transferred to the growth chamber filled with vermiculite maintained at 28°C/18°C (day/night) with a 12 h photoperiod (photosynthetically active radiation = 400 μmol/m^2^/s). Cold stress condition maintained at 10°C/ 8°C (day/night), other conditions remain unchanged. Control, T1, T2, T3, T4, and T5 are the treatment of 0, 1, 10, 50, 100, and 1,000 μM sodium selenite (Sinopharm Chemical Reagent Co., Ltd), respectively. The concentration of exogenous application of sodium selenite was 1 μM under cold stress and normal conditions in the follow-up trial. The concentrations of melatonin (Tokyo Chemical Industry) and CPA (Tokyo Chemical Industry) were 1 μM each.

### Determination of Plant Growth Index

After 10 days of treatment, the fresh shoot weights of cucumber seedlings were measured. Plant height was the distance from the bottom of the stem to the apical meristem. They were measured using a ruler. SPAD value was measured at the third leaf from the apical meristem after 5 and 10 days treatments using SPAD-502 Plus meter, with 5 replicates for each treatment.

### Element Content Measurement

For analysis of element contents of N, P, and K, the roots of each seedling in the same treatment of five plants were taken, and then were rinsed with deionized water and dried at 70°C to a constant weight, leaves of 5 plants in one pot were mixed together, which was considered as one replicate. There were three replicates for each treatment. After pulverizing, a mixture of 0.2 g of powdered dry cucumber leaf was digested in a solution of H_2_SO_4_-H_2_O_2_, and the extract was used to determine N, P, and K content. N was determined by the Kjeldahl method and P was determined by vanadomolybdate colorimetric procedure (Wang et al., 2010; Gong et al., 2013). K was determined by a flame photometer (Wang and Zhao, 1995).

### The Net Photosynthetic Rate Analysis

The net photosynthetic rate (Pn) was determined on the third fully expanded leaves by a photosynthesis system (LI-6400, Lincoln, United States). The measurement was performed after the treatments, at 5 d and 10 d between 9:00 and 11:00 A.M. while maintaining the air temperature CO_2_ concentration, and PPFD at 25°C, 400 μmol/m^2^/s, and 1,000 μmol/m^2^/s, respectively.

### Transmission Electron Microscopy Analysis

The transmission electron microscopy analysis of chloroplast ultrastructure was carried out as described by Fukuda et al. (2013). Samples were taken from the third leaves, counting from the tip stem, of control, selenium application, cold stress, and selenium application under cold stress conditions after treatment for 10 days. Samples were harvested at 9:00 to 11:00 A.M. Samples were rapidly cut into 1-mm × 1-mm squares and inserted into 0–4°C pre-cooled fixative within 1 min. The ratio of the sample and the liquid was 1:20–40. Every 100 mL fixative contained 10 mL 25% glutaraldehyde, 50 mL 0.2 M phosphate buffer (pH 7.4), and 40 mL double-distilled water. The samples were air exhausted in a vacuum after being immersed in the fixative liquid. The tissues were washed using 0.1 M PB (pH 7.4) for three times, 15 min each. Post-fix: Tissues were post-fixed with 1% OsO_4_ in 0.1 M PB (pH 7.4) for 7 h at room temperature under the condition of light avoided. After removing from OsO_4_, the tissues were rinsed in 0.1 M PB (pH 7.4) three times, 15 min each. Dehydrated at room temperature as follows: 30% ethanol for 1 h; 50% ethanol for 1 h; 70% ethanol for 1 h; 80% ethanol for 1 h; 95% ethanol for 1 h; 100% ethanol for 1 h; 100% ethanol for 1 h; ethanol:acetone=3:1 for 0.5 h; ethanol:acetone = 1:1 for 0.5 h; ethanol:acetone = 1:3 for 0.5 h; and pure acetone for 1 h. Resin penetration and embedding as follows: acetone:EMBed 812 = 3:1 for 2–4 h at 37°C; acetone:EMBed 8121 = :1 overnight at 37°C; acetone:EMBed 812 = 1:3 for 2–4 h at 37°C; pure EMBed 812 for 5–8 h at 37°C; poured the pure EMBed 812 into the embedding models and inserted the tissues into the pure EMBed 812, and then kept in 37°C oven overnight. Polymerization: The embedding models with resin and samples were moved into 65°C oven to polymerize for more than 48 h. And then the resin blocks were taken out from the embedding models for standby application at room temperature. Ultrathin section: The resin blocks were cut to 60–80 nm thin on the ultramicrotome (Leica UC7, Leica), and the tissues were fished out onto the 150-mesh cuprum grids with formvar film. Stained with 2% uranium acetate saturated alcohol solution for 8 min under the condition of avoid light, rinsed with 70% ethanol for 3 times and then with ultra-pure water for 3 times. Lead citrate (2.6%) stained for 8 min by avoiding CO_2_, and then rinsed with ultra–pure water three times. After drying by the filer paper, the cuprum grids were put into the grids board and dried overnight at room temperature. Observation and image capture: The cuprum grids were observed under transmission electron microscope (HT7800/HT7700, HITACHI) and images were taken.

### Analysis of Reactive Oxygen Species Accumulation

The histochemical staining of ${\text{O}}_{2}^{-}$ was performed using nitroblue tetrazolium (NBT) according to Xia et al. (2009). For the histochemical staining of ${\text{O}}_{2}^{-}$, leaves were vacuum-infiltrated with 0.1 mg/mL NBT in 25 mM K-Hepes buffer (pH 7.8) and cultivated at 25°C in the dark for 2 h. Leaves were rinsed in 80% (v/v) ethanol for 20 min at 70°C, mounted in lactic acid/phenol/water (1:1:1; v/v/v), and photographed.

### Determination of Enzymatic Activity

Three hundred milligrams of cucumber leaves were ground with 3 mL of cold 50 mM phosphate buffer solution buffer (pH 7.8), which included 0.2 mM ethylenediaminetetraacetic acid (EDTA), 2 mM ascorbate, and 2% polyvinylpyrrolidone (PVP). The homogenates were centrifuged at 4°C for 20 min at 12,000 g and the supernatants were used for the determination of antioxidant enzymatic activities. Superoxide dismutase (SOD) activity was assayed by measuring its ability to inhibit the photochemical reduction of nitro blue tetrazolium (Stewart and Bewley, 1980). Catalase (CAT) activity was measured as the decrease in absorbance at 240 nm because of the decrease in H_2_O_2_ extinction (Chamnongpol et al., 2010). Ascorbate peroxidase (APX) activity was measured by the decrease in absorbance at 290 nm as the ascorbic acid (ASA) was oxidized (Durner and Klessig, 1995). Peroxidase (POD) activity was measured as the increase in absorbance at 470 nm because of guaiacol oxidation (Nickel and Cunningham, 1969).

### Quantification of Melatonin

According to Byeon and Back (2014) and (Yan et al., 2019b), 0.2 g samples were ground to a powder in liquid nitrogen and then extracted with 1.5 mL methanol at 4°C for 30 min. After centrifugation of the extraction mixture for 5 min at 8,000 g, the supernatant was taken. The precipitation was repeatedly extracted once. The two extracts were combined, then filtrated with 0.22 μm filter membrane, evaporated to dryness and dissolved in 0.2 mL of 40% methanol. Aliquots of 10 μL were subjected to HPLC using a fluorescence detector system (Waters). The samples were separated on a Sunfire C18 column (4.6 × 150 mm; Waters) and the mobile phase constitution was water:methanol = 6:4. The flow rate was 1 mL/min. Melatonin was detected at 286 nm excitation and 352 nm emission wavelengths. The melatonin was eluted at 8.9 min under these conditions. The standard curve was Y=963.3X+0.7044; R = 0.9999, with the melatonin concentrations of 0.03, 0.05, 0.1, 0.5, 1, 5 μg /mL were used.

### Real-Time Quantitative PCR Analysis

Total RNA was extracted from cucumber leaves using the TRIzol method according to the supplier's instructions (Invitrogen, Carlsbad, CA, United States). DNase was used during RNA extraction to reduce DNA contamination. cDNA synthesis was performed according to standard procedures of a Revert Aid First Strand cDNA synthesis kit (Fermentas, Ontario, Canada). The cucumber actin gene was used as the internal control for the quantification of transcripts. Real-time quantitative PCR using an aliquot of cDNA (1/500), Power SYBR Green PCR Master Mix (*ABI*), and 200 nM each primer on an ABI Prism 7900 HT machine. Data were analyzed using SDS 2.500 software (*ABI*), and relative expression was calculated using the comparative cycle threshold method with normalization of data to the geometric average of the internal control genes (Pfaffl, 2001).

The primers of *TDC* (*Csa3G611340*; F: 5′-ACCATCGTCGTCTTCGTTATC-3′ and R: 5′-CATTTCTCTGCTCGGACTTCT-3′), *T5H* (*Csa6G501350*; F: 5′-GCCTGGTTCACACCATCATA-3′ and R: 5′-ATGCTGGAAGTGTGGATTAGG-3′), *SNAT* (*Csa4G336250*; F: 5′-CGGGTAGCTGAAGAAGAAGAAG-3′ and R: 5′-AAATGGCCGGAGCAAAGA-3′), *COMT* (*Csa4G091880*; F: 5′-TCCGACCATTCCACCATTAC-3′ and R: 5′- CCGACATCCACCACTGAATTA-3′), and *ACTIN* (*Csa6G484600*; F:5′-CAGGAACTTGAGACTGCTAAGA−3′ and R: 5′-CGATGAGAGATGGCTGGAATAG-3′). Primer search method: the protein encoded by the gene in *Arabidopsis thaliana* was obtained by comparing tomato genes in the literature (Xu, 2016; Ahammed et al., 2018). Gcorn plant (http://www.plant.osakafu-u.ac.jp/~kagiana/gcorn/p/19/) was used to find the protein encoded by the direct homologous gene of this protein in cucumber, and then compared in cucumber genome to obtain the most similar gene. Tomato Genome website: https://solgenomics.net/tools/blast. Cucumber Genome website: http://cucurbitgenomics.org/. Arabidopsis Genome website: https://www.arabidopsis.org/.

### Statistical Analysis

Data were plotted using Microsoft Excel 2010 software. Data were presented as the mean ± standard deviation of three replicates (five plants in each replicate). Statistical analyses were carried out by ANOVA using SAS software. Differences between treatments were determined by the least significant differences with *p* < 0.05.

## Results

### The Dose-Dependent Curves for Plant Growth in Response to Exogenous Selenium

**Figure 1:**
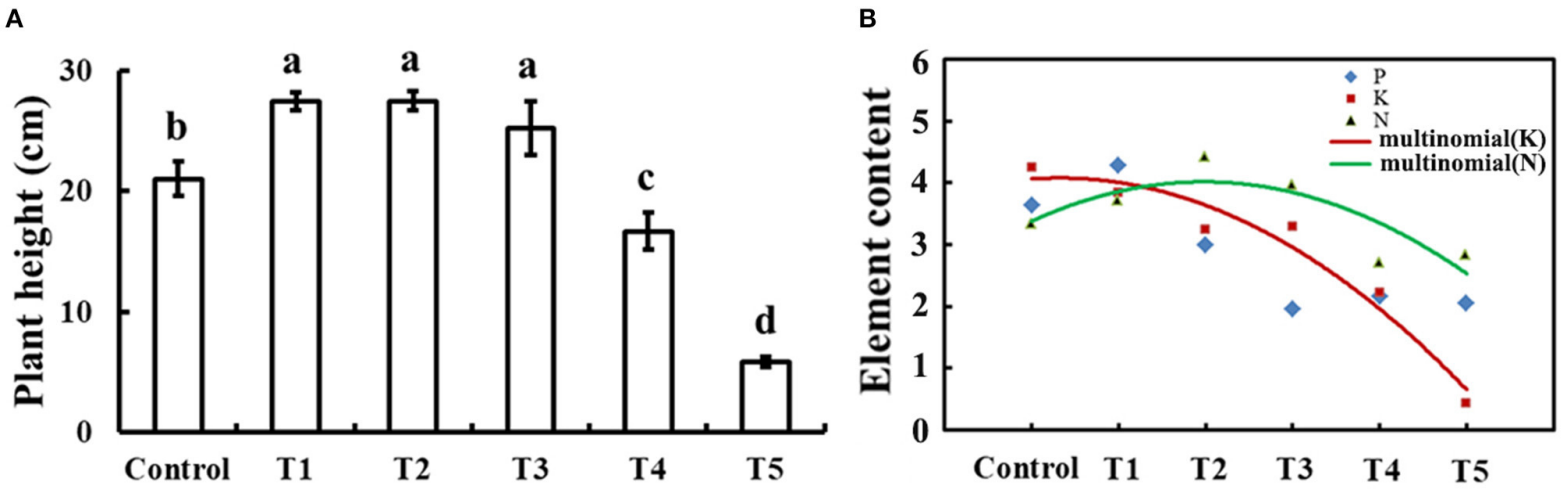
Plant height and element contents in response to exogenous selenium with different concentrations. **(A)** Plant height of cucumber seedlings treated with 0, 1, 10, 50, 100, and 1,000 μM exogenous selenium for 10 days. These values are expressed as means of three replicates ± SD, and different letters are significantly different (*P* < 0.05). **(B)** Element (N, P, and K) content with 0, 1, 10, 50, 100, and 1,000 μM exogenous selenium for 10 days in cucumber seedlings root. These values are expressed as means of three replicates.

To determine the appropriate level of selenium for cucumber seedlings growth, plant height and element content of 30-day-old cucumber seedlings on 0, 1, 10, 50, 100, and 1,000 μM sodium selenite treatments for 10 days were compared as shown in Figure 1. Compared with no selenium treatment, treatment with exogenous selenium significantly enhanced plant height within the concentrations applied from 1 to 50 μM sodium selenite (*p* < 0.05). However, 100 and 1,000 μM sodium selenite treatments significantly inhibited plant growth (Figure 1A). A dose-dependent response was observed with low concentrations of selenium promoting plant growth and high concentrations inhibiting plant growth. Based on the different concentrations of selenium treatments in cucumber seedling root, results showed that the relative content of nitrogen presented a trend of rise and then decline, and in 10 μM sodium selenite of T2 treatment, the nitrogen content peaked. 1, 10, and 50 μM concentrations of sodium selenite promoted the cucumber seedling root nitrogen accumulation. The relative contents of potassium decreased with the content of sodium selenite increased. Phosphorus showed a trend of rise, then reduced, and the final leveling off, and 1 μM sodium selenite treatment showed phosphorus accumulated in cucumber seedling roots the most. Fitting the trend line of nitrogen and potassium, the trend lines on the intersection under 1–10 μM sodium selenite treatments, and meet 1 μM sodium selenite treatments closer. Under the condition of no significant difference in plant growth, the higher root nutrient element contents, the more plant growth potential (Kulcheski et al., 2015). According to a comprehensive estimation for these parameters, the most powerful concentration of selenium (1 μM) was used for further studies.

### The Response of Exogenous Selenium to Cold Tolerance of Cucumber Seedlings

**Figure 2:**
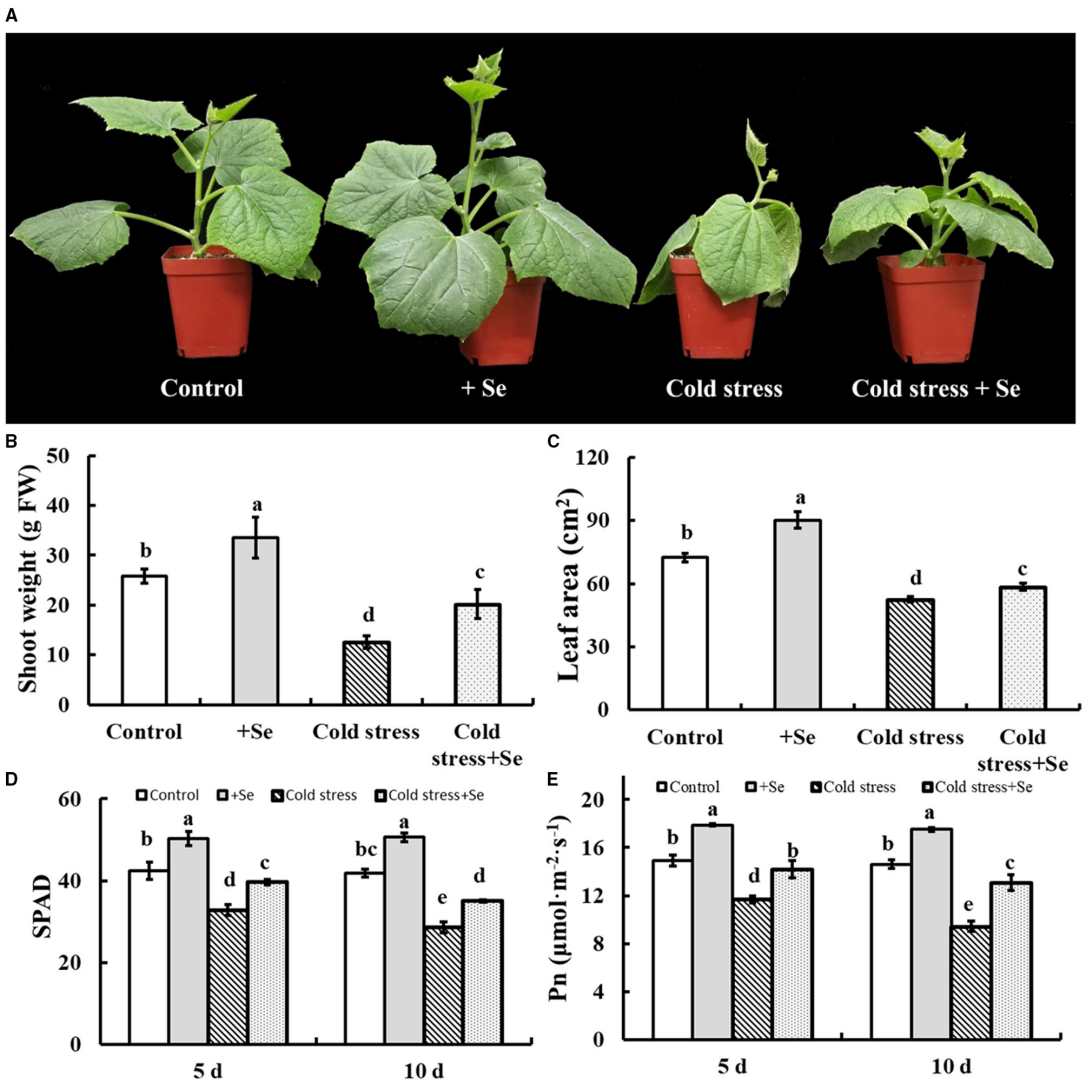
Effects of exogenous selenium on cold stress of cucumber seedlings. **(A)** The phenotype of control, selenium application, cold stress, and selenium application under cold stress. **(B)** Shoot weight of control, selenium application, cold stress, and selenium application under cold stress. **(C)** Leaf area of control, selenium application, cold stress, and selenium application under cold stress. **(D)** SPAD value of control, selenium application, cold stress, and selenium application under cold stress with 5 and 10 days treatments, respectively. **(E)** Net photosynthetic rate of control, selenium application, cold stress, and selenium application under cold stress with 5 and 10 days treatments, respectively. Values are expressed as means of three replicates ± SD, and different letters are significantly different (*P* < 0.05).

Figure 2A shows that 1 μM sodium selenite treatment significantly alleviated cold tolerance of cucumber seedlings, shoot weight, and leaf area had been detected (Figures 2B,C). Selenium application significantly increased leaf size and shoot fresh weight of cucumber seedlings. Not only that, but selenium application significantly alleviated the decrease of leaf size and shoot fresh weight caused by cold stress. Thus exogenous selenium application significantly alleviated the inhibition of cold stress on cucumber seedling growth. Under normal culture conditions, the SPAD value and the net photosynthetic rate of cucumber leaves were significantly increased by selenium application (Figures 2D,E). Exogenous selenium application significantly increased the SPAD value of cucumber leaves compared with no selenium application (Figure 2D). Compared with 5 days of cold stress, SPAD value of 10 days of cold stress decreased significantly. The net photosynthetic rate of cucumber leaves decreased significantly under cold stress, and the net photosynthetic rate of cucumber leaves under 10 days of cold stress was significantly lower than that of the leaves under 5 days of cold stress.

### Exogenous Application of Selenium Protected the Photosynthetic Apparatus and Alleviated the Oxidative Stress Induced by Cold Stress

**Figure 3:**
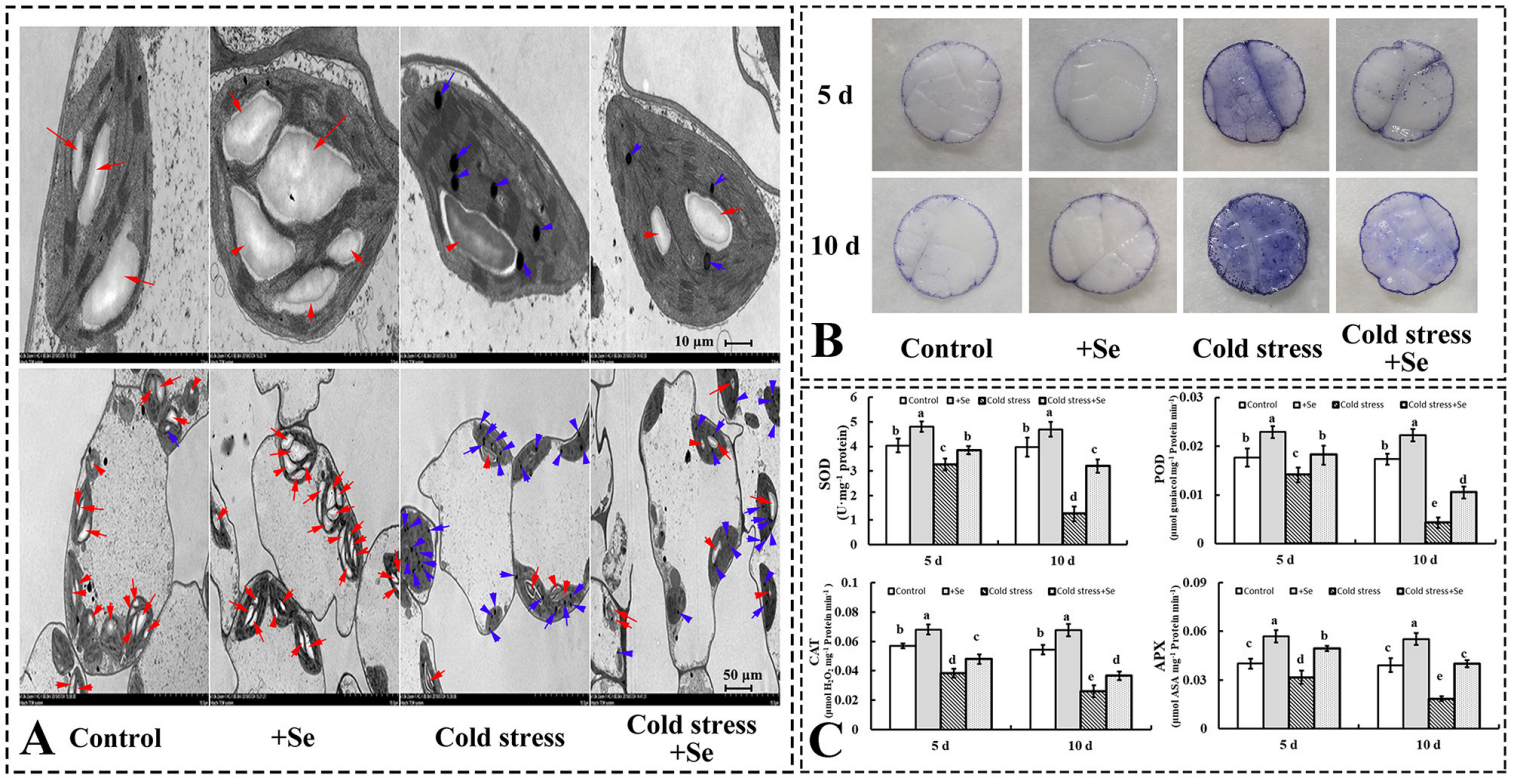
Exogenous application of selenium protected the photosynthetic apparatus and alleviated the oxidative stress induced by cold stress. **(A)** Ultrastructures of cells of cucumber seedlings leaf of control, selenium application, cold stress, and selenium application under cold stress. Red arrow points to starch grain, blue arrow points to osmiophilic granule. **(B)** ${\text{O}}_{2}^{-}$ in leaves was detected by NBT staining. **(C)** The activity levels of SOD, POD, CAT, and APX of control, selenium application, cold stress, and selenium application under cold stress with 5 and 10 days treatments, respectively. Values are expressed as means of three replicates ± SD, and different letters are significantly different (*P* < 0.05).

As a photosynthetic apparatus, chloroplast ultrastructures of cucumber leaves were significantly affected by exogenous application of selenium compared with control conditions. Exogenous selenium application increased the number and the size of starch grains (Figure 3A). However, cold stress decreased the number of starch grains and increased the number of osmiophilic granules. Under cold stress condition, exogenous application of selenium increased starch grains compared with no selenium application treatment under cold stress condition (Figure 3A). Histochemical observation of ${\text{O}}_{2}^{-}$ in leaves using NBT staining corroborated the biochemical analysis, indicating that the accumulation of reactive oxygen species (ROS) was significantly increased, especially under cold stress condition after 10 days. However, the exogenous application of selenium increased the ROS-scavenging capability in cold stressed cucumber leaves (Figure 3B). The activities of the investigated ROS scavenging-related enzymes were greater in the treatment of selenium application than no application of selenium under cold stress (Figure 3C). Under control conditions, some enzymes, such as SOD, POD, CAT, and APX, also showed greater activity levels in the treatment of exogenous selenium application (Figure 3C).

### Effects of Exogenous Selenium on the Endogenous Melatonin

**Figure 4:**
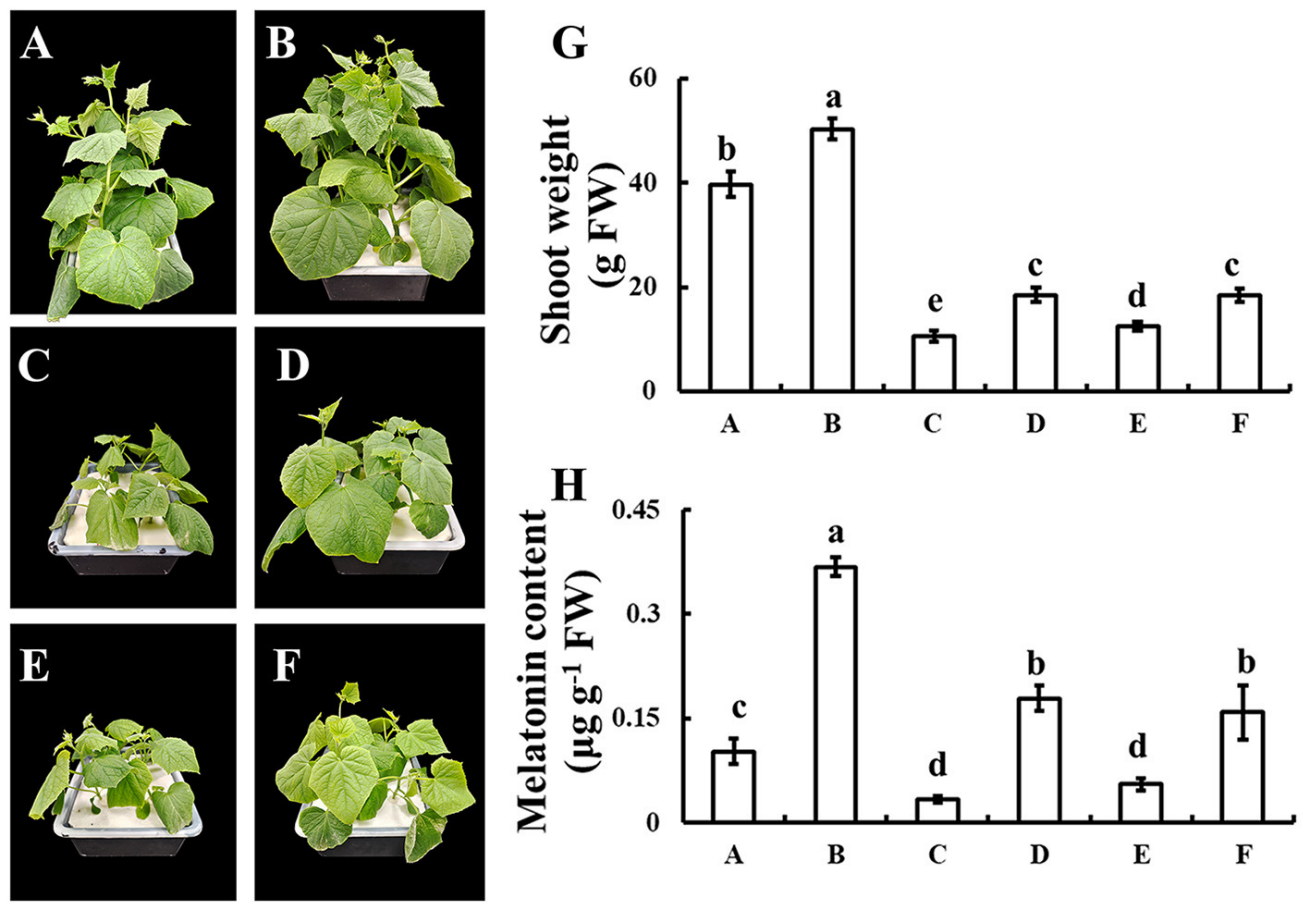
Effects of exogenous selenium on endogenous melatonin. **(A–D)** The phenotype of control, selenium application, cold stress, and selenium application under cold stress condition. **(E)** The phenotype of selenium and melatonin biosynthesis inhibitor application under cold stress condition. **(F)** The phenotype of selenium, melatonin, and melatonin biosynthesis inhibitor application under cold stress condition. **(G)** Shoot weight of A-F. **(H)** Melatonin content of A-F. Values are expressed as means of three replicates ± SD, and different letters are significantly different (*P* < 0.05).

To discover whether melatonin participates in selenium-enhanced cold tolerance of cucumber seedlings, the pharmacologic method was used. Application of selenium significantly increased the growth of cucumber seedling, which was shown as the shoot weight significantly increased with selenium application compared with normal condition (Figures 4A,B,G). Under cold stress condition, plant shoot weight decreased significantly, whereas plant shoot weight loss reduced when treated with selenium (Figures 4C,D,G). Treatment with both selenium and CPA showed only a partial alleviation of cold stress effects (Figures 4D,E,G). Adding selenium, melatonin, and melatonin biosynthesis inhibitor under cold stress condition restored phenotype to only treated with selenium under cold stress condition (Figures 4D,F,G).

### Genes Expression Related With Melatonin in Cucumber Seedlings

**Figure 5:**
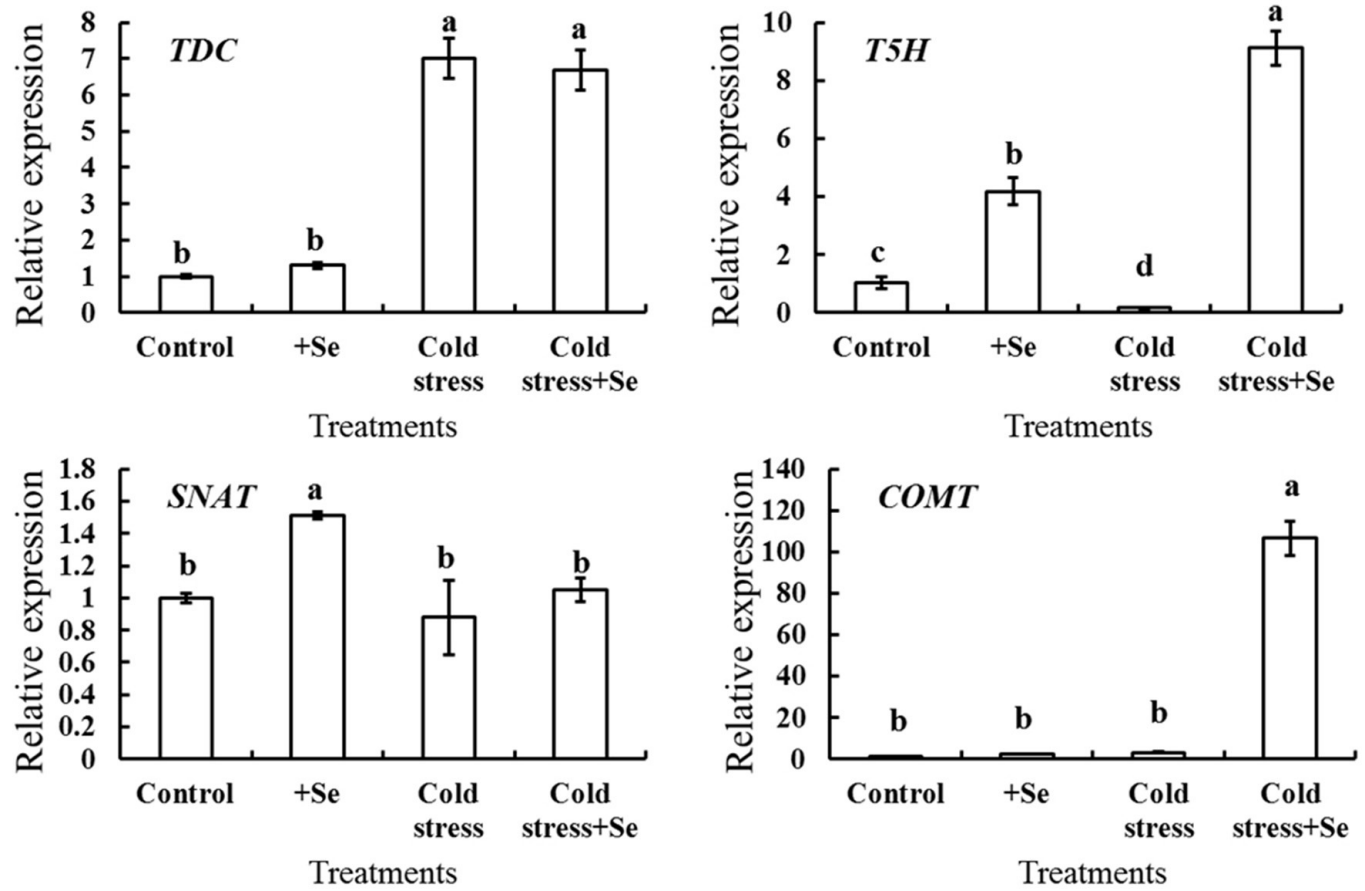
Relative expression of melatonin biosynthesis genes in response to selenium treatment. RT-QPCR analyses was used to assess the relative expression of *TDC, T5H, SNAT*, and *COMT* in cucumber seedlings leaf of control, selenium application, cold stress, and selenium application under cold stress. Values are expressed as means of three replicates ± SD, and different letters are significantly different (*P* < 0.05).

It was demonstrated that melatonin plays a vital role in enhancing cold tolerance by the application of exogenous selenium. A series of genes related to melatonin biosynthesis was analyzed by RT-QPCR to detect the expression of those genes that showed more response to selenium under cold stress condition (Figure 5). The expression of *T5H* and *SNAT* increased after the application of selenium under normal conditions. However, under cold condition, application of selenium significantly increased the expression of *T5H* and *COMT*. These indicated that selenium induced higher expression of *T5H*, with the effect further enhanced by the cold stress condition. The expression of *COMT* increased even hundredfold with the application of selenium under cold stress condition compared with normal conditions. Figure 5 also shows that the expression of *TDC* increased significantly under cold stress condition, while there seemed to be no significant changes with selenium application neither under cold stress nor under normal conditions. These suggested that *T5H* and *COMT* might be vital in enhancing melatonin content with selenium application under cold stress condition.

## Discussion

Selenium has been studied in plants undergoing adaptation to unfavorable stresses and fruit quality (Zhu et al., 2018; Wen, 2021). A previous study showed that the treatments with 0.5 and 1.0 mg/kg Se significantly increased biomass and chlorophyll content of wheat seedlings (Chu et al., 2010). Approximately 5 mg/L of sodium selenite solution had the greatest stress-alleviating effects that assist in protecting strawberry seedlings in a low-temperature environment (Huang et al., 2018). Cucumber, as a warm-loving vegetable, is vulnerable to cold stress in winter and spring. No studies have shown that the exogenous application of selenium enhances cold tolerance of cucumber, let alone explore its application concentration. Because of the difference in selenium concentration apply to different crops, the article reported that 1–50 μM selenium concentration significantly increases the plant height of cucumber seedlings under normal conditions. Meanwhile, through the determination of root element contents of cucumber seedlings, it was determined that the content of N, P, and K in the root system was optimal under the treatment of 1 μM selenium. The high content of N, P, and K in the root system not only indicated that the overall development of the root system was good, but also indicated that the root system had a good potential to transport nutrients to the shoot in the future. When there was no significant difference in the shoot height, the comparison of element content in the root system could effectively distinguish the subsequent development direction and difference in plants.

Hence, 1 μM selenium was used and our study also showed that 1 μM selenium effectively enhances cold tolerance of cucumber seedlings. This study showed that the exogenous selenium effectively reduced the inhibition on shoot weight of cucumber seedlings under cold stress condition (Figure 2B). The accumulation of fresh shoot weight depends on plant photosynthesis (Wen et al., 2021), and selenium effectively reduced the inhibition of the net photosynthetic rate of a plant under cold stress. On the one hand, the application of selenium effectively increased the leaf area of functional leaves of cucumber seedlings under cold stress condition, and then increased the photosynthetic area of the plant. On the other hand, selenium application significantly increased SPAD value, chlorophyll content, and net photosynthetic rate of leaves under the same leaf area under cold stress condition. Interestingly, there was no significant difference in the SPAD value of cucumber leaves under 5 days of cold stress and 10 days of cold stress when exogenous selenium was applied, and the net photosynthetic rate of cucumber leaves under 10 days of cold stress was significantly higher than that under 5 days of cold stress. These results indicated that selenium application significantly enhanced the increase of net photosynthetic rate under cold stress, and the increase of selenium not only increased the content of chlorophyll in leaves but also involved other regulatory pathways.

Because exogenous selenium application significantly affected the photosynthesis of plants under cold stress, transmission electron microscopy was used to observe the subcellular structure of cucumber seedling functional leaves (Figure 3A). Starch grains representing nutrient accumulation decreased significantly under cold stress, but the exogenous application of sodium selenite could reduce the decrease of starch grains. Stress easily leads to photoelectron transfer spilt and produces ROS (Wen et al., 2019). The detection of ${\text{O}}_{2}^{-}$ showed that exogenous application of selenium reduced the content of superoxide anion. It was found that exogenous selenium could effectively improve the activity of antioxidant enzymes, and then reduce the production of ROS that damage the cells. Therefore, exogenous selenium application promoted the accumulation of organic matter produced by photosynthesis while also reducing the damage caused by the generation of ROS to cells under cold stress condition.

Melatonin plays an important role in plant resistance (Li et al., 2016; Wen et al., 2016; Gong et al., 2017; Yan et al., 2019a). Melatonin maintained cell membrane stability, increased antioxidant enzymes activities, improved the process of photosystem II, and induced alterations in Bermudagrass and rice metabolism under cold stress (Fan et al., 2015; Han et al., 2017). It has been reported that exogenous melatonin alleviates cold stress by upregulating the expression of C-repeat-binding factors, a cold-responsive gene, *COR15a*, et al., and stimulate the biosynthesis of cold-protecting compounds (Bajwa et al., 2014). There are also reports that melatonin enhances cold tolerance by regulating energy and proline metabolism (Liu et al., 2020). Most studies focused on melatonin alleviating plant cold stress, and our studies reported that melatonin as a downstream signal was involved in the improvement of exogenous selenium application by enhancing tolerance of cucumber seedlings under cold stress condition (Figure 4). The present study provided several lines of evidence that melatonin, as a downstream signal, was involved in selenium enhanced cold tolerance of cucumber seedlings. First, selenium increased the content of melatonin, both under normal and cold stress conditions. Second, the alleviating effect of exogenous selenium application on cold stress was significantly inhibited by melatonin synthesis inhibitors. Third, the expression of melatonin biosynthesis genes *T5H* and *COMT* increased significantly with selenium treatment under cold stress of cucumber seedlings, which might lead to the increase of melatonin content. It has been reported in tomato plant that *COMT* silencing aggravates heat stress leading to the reduction in photosynthesis (Ahammed et al., 2018), which confirms our results in another way. At the same time, these give the hypotheses, whether the combination application, the precursor of melatonin tryptophan and selenium, effectively improves the cold tolerance of cucumber seedlings, which has important reference and theoretical support for the application of improving plants' cold tolerance in agriculture, needs to be verified by more experiments.

**Figure 6:**
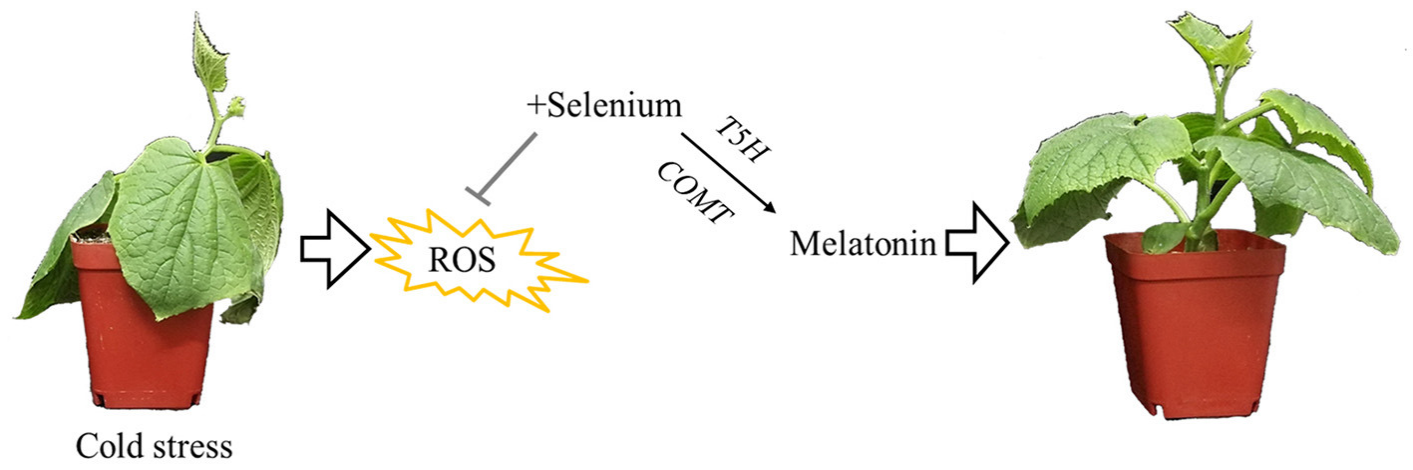
Schematic illustration for melatonin participated in enhancing cold tolerance by exogenous application of selenium. Cold stress inhibited plant growth and increased the production of reactive oxygen species; however, selenium reduced cold stress damage to the plant. Meanwhile, melatonin as the downstream signaling participated in selenium-enhanced cold tolerance of cucumber seedlings. Melatonin biosynthesis genes *T5H* and *COMT* play vital roles in exogenous selenium application enhancing the melatonin content, promoting plant cold tolerance.

Based on our results and analyses, a schematic illustration of a possible mechanism for melatonin involvement in enhancing cold tolerance by exogenous selenium application in cucumber seedlings was prepared as seen in Figure 6. Cold stress inhibited plant growth and increased the production of ROS; however, selenium reduced cold stress damage to the plant. Meanwhile, melatonin as the downstream signaling participated in selenium-enhanced cold tolerance of cucumber seedlings. Melatonin biosynthesis genes *T5H* and *COMT* play vital roles in exogenous selenium application enhancing melatonin content promoting plant cold tolerance. However, research on selenium and melatonin signals in plants is just at the beginning, and more work is needed to gain a more accurate understanding of the signal pathway in cold tolerance regulation.

## Data Availability Statement

The raw data supporting the conclusions of this article will be made available by the authors, without undue reservation.

## Author Contributions

DW, NY, and KS: conceived and designed the research. NY and XK: performed the research. NY and DW: analyzed the data. KS, XW, KW, and JG: contributed materials/analysis tools. NY: wrote the first draft of the manuscript. DW: improved the first draft of the manuscript. All the authors have read and approved this manuscript.

## Funding

This research was supported by the Agriculture Industrial Technology System Funding of Shandong Province of China (Grant Number SDAIT-05-07) and the Agricultural Scientific and Technological Innovation Project of Shandong Academy of Agricultural Sciences (Grant Numbers CXGC2016B06, CXGC2018E08, and CXGC2021A22).

## Conflict of Interest

The authors declare that the research was conducted in the absence of any commercial or financial relationships that could be construed as a potential conflict of interest.

## Publisher's Note

All claims expressed in this article are solely those of the authors and do not necessarily represent those of their affiliated organizations, or those of the publisher, the editors and the reviewers. Any product that may be evaluated in this article, or claim that may be made by its manufacturer, is not guaranteed or endorsed by the publisher.
